# First-principles design of nanostructured hybrid photovoltaics based on layered transition metal phosphates

**DOI:** 10.1038/s41598-017-01296-0

**Published:** 2017-04-28

**Authors:** Levi C. Lentz, Alexie M. Kolpak

**Affiliations:** Department of Mechanical Engineering, Massachusetts Institute of Technology, 77 Massachusetts Ave, Cambridge, MA 02139 USA

## Abstract

The performance of bulk organic and hybrid organic-inorganic heterojunction photovoltaics is often limited by high carrier recombination arising from strongly bound excitons and low carrier mobility. Structuring materials to minimize the length scales required for exciton separation and carrier collection is therefore a promising approach for improving efficiency. In this work, first-principles computations are employed to design and characterize a new class of photovoltaic materials composed of layered transition metal phosphates (TMPs) covalently bound to organic absorber molecules to form nanostructured superlattices. Using a combination of transition metal substitution and organic functionalization, the electronic structure of these materials is systematically tuned to design a new hybrid photovoltaic material predicted to exhibit very low recombination due to the presence of a local electric field and spatially isolated, high mobility, two-dimensional electron and hole conducting channels. Furthermore, this material is predicted to have a large open-circuit voltage of 1.7 V. This work suggests that hybrid TMPs constitute an interesting class of materials for further investigation in the search for achieving high efficiency, high power, and low cost photovoltaics.

## Introduction

Photovoltaic (PV) devices based on organic or hybrid organic-inorganic materials are of interest because of their low cost, low environmental impact, and structural flexibility, among a range of other scientific and engineering applications. However, current organic and hybrid PV devices have relatively low efficiencies compared to state-of-the-art silicon PV due to high exciton recombination rates and/or low open-circuit voltage (*V*_oc_). In general, the former is due to trap states within the material as well as interfacial recombination centers, while the latter is a result of non-optimal band alignment.

Bulk heterojunctions (BHJs), composed of donor and acceptor molecules, are one approach to addressing these challenges. In BHJs, deposition is focused on maximizing the surface area between the donor and acceptor molecules to increase the probability of charge separation. Further, manufacturing methods allow for thin film deposition, decreasing overall feature lengths. However, the atomic-scale disorder at the donor-acceptor junctions often leads to high densities of interfacial states that act as recombination centers. These mid-gap states may also lead to a decrease in the overall *V*_OC_. In this work we present a framework that utilizes layered materials to leverage existing organic molecules in a way that minimizes recombination centers and maximizes both exciton separation and *V*_OC_.

Previous efforts to increase *V*_OC_ in BHJs have generally focused on two approaches: (i) controlling morphology, often by employing carbon nanotube scaffolds^1,2^ or nanostructured surfaces^3,4^ and/or (ii) incorporating multiple acceptor layers to optimize band alignment between donor, acceptor, and electrode^5^. Efficiency gains have also been achieved using ferroelectric materials that may create a net dipole between the electrodes of the device^6,7^. The improvements due to these approaches, however, may be offset by atomic-scale disorder that leads to high exciton recombination rates at the various interfaces within the device.

To address these issues, we propose a new class of nanostructured hybrid organic-inorganic photovoltaics composed of layered transition metal phosphates (TMPs) covalently bound to organic absorber molecules to form nanostructured superlattices. Crystalline, layered TMP superlattices of this type have been previously synthesized using solution-based processing methods^8^, but to date have not been widely investigated in the context of electronic or optical applications. Our recent work suggests high tunability of TMP band energies through simple surface functionalization^9^. Using these relationships, we optimize a new hybrid TMP-organic superlattice based on zirconium phosphate, α-Zr(HPO_4_)_2_, one of the most well-studied layered TMPs, and we show that this new material is predicted to exhibit very low exciton recombination, high electron and hole mobilities, and a large open-circuit voltage of 1.7 eV.

## Approach

### Nanostructured PV Design

**Figure 1:**
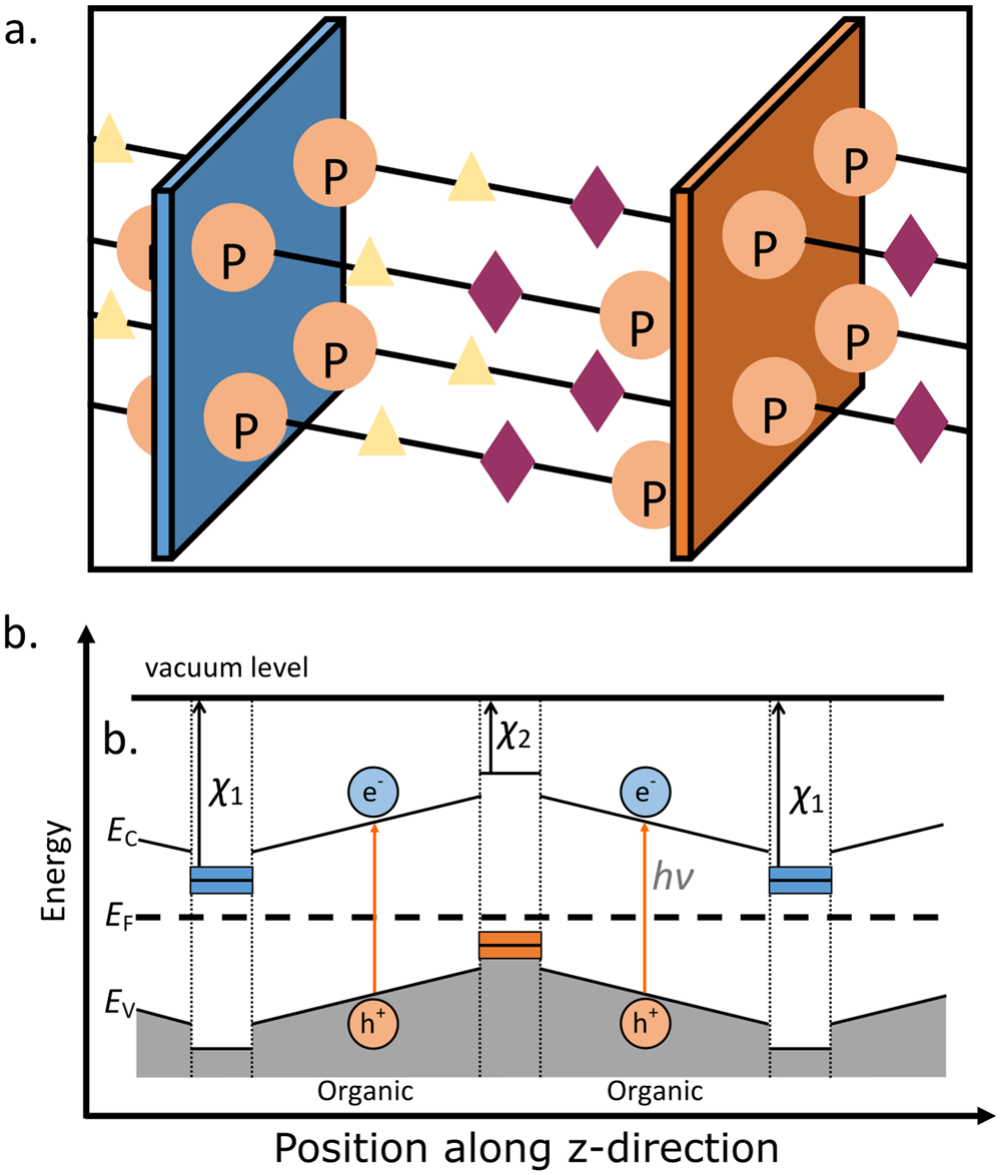
(**a**) Schematic of the proposed atomic structure, in which alternating inorganic hole and electron transport layers (blue and orange sheets, respectively) are separated by polar organic absorber molecules. Triangles and diamonds represent organic functional groups chosen to tune the polarization and the band alignment. (**b**) Schematic band energy diagram corresponding to the structure in (**a**). *χ*_1_ and *χ*_2_ are the electron affinities of the inorganic electron and hole conducting layers, respectively.

The proposed material architecture is illustrated schematically in Fig. 1. In this architecture, planar organic molecules link 2D inorganic sheets with nanoscale separation. The 2D inorganic sheets have large band gaps, so excitons are generated only within the organic regions. As shown in the schematic band diagram in Fig. 1b, these excitons are separated via an electric field, which is determined by a combination of the dipole of the polar organic molecule and the electron affinity differences between the inorganic layers. The characteristic length scale over which this dipole acts (the distance between adjacent inorganic sheets) is on the order of 1–10 nanometers. Small length scale polarity such as this has been demonstrated in the surface functionalization of 2D sheets to dissociate water molecules^10^. This length scale enables large local electric fields as well as transit length scales that are significantly shorter than the mean free paths of the excitons or the separated charge carriers in typical organic materials. Coupled with appropriate design of the energy band alignment, this ensures that carriers are transferred into the relevant inorganic transport layers, from which there is a significant energy barrier to escape, as illustrated in Fig. 1b. Thus, electrons and holes in the inorganic transport channels have a negligible probability for recombination.

This hypothetical framework may, in principle, be achieved with many different materials combinations. We choose to implement this design using layered TMPs, which have been demonstrated to self-assemble into ordered crystal structures with covalently bound organic materials. As discussed below, the range of both layered TMP compounds and polar organic absorber molecules provides potential for a wide range of materials properties.

### Materials Platform

Similar to graphite and transition metal chalcogenides such as MoS_2_, bulk layered TMPs are composed of stacks of two-dimensional (2D) crystalline sheets bound primarily via van der Waals interactions along the stacking direction. Solution-based syntheses of TMPs have been demonstrated for a range of chemical compositions^8^ to form ordered, crystalline superlattices composed of alternating inorganic and organic layers with nanoscale spacing^11–14^. Experimental measurements of zirconium phosphate show that bulk conductivity can tuned from about 0.001 to 1 S/cm^15^ via intercalation of cations such as Li, Na, and K between the 2D sheets. This conductivity is comparable to or greater than that of many organic materials used in BHJs, such as PEDOT:PSS^16^, ITO:PET^17^, and P3HT:PCBM^17^.

**Figure 2:**
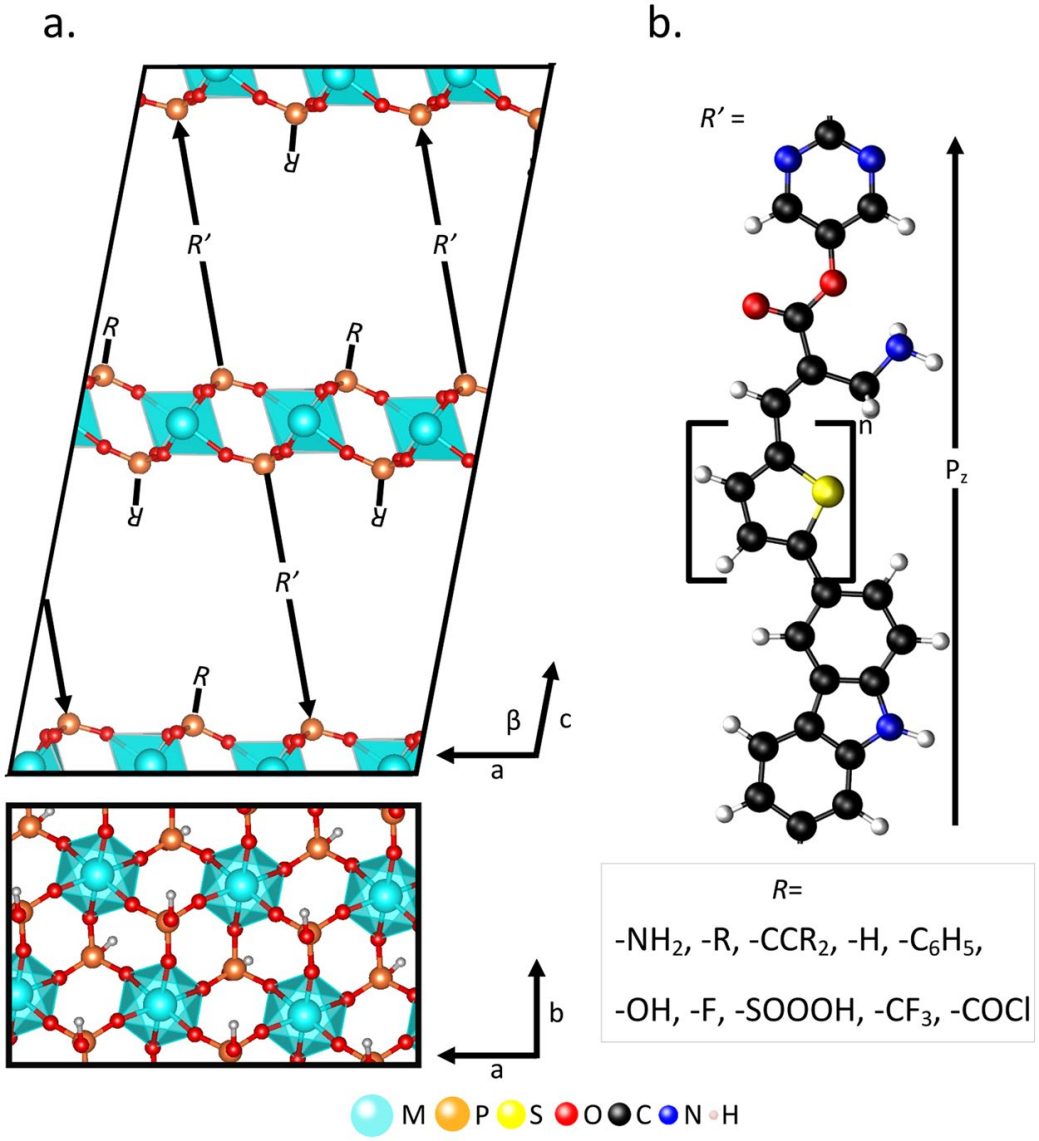
Schematic illustrating the range of structures studied in this work. (**a**) Top, side view of supercell with R’ molecules. Arrows show the direction of molecular dipoles. Bottom, in-plane view of TMP with R = -OH. (**b**) Representative molecule of R’ studied in this work.

In this work we focus on materials based on α-Zr(HPO_4_)_2_^18^, as illustrated in Fig. 2, with the general composition Zr_2−x_*M*_x_(*RR*^’^PO_3_)_4_, where *M* is a transition metal substitution at the Zr lattice position, *R’* is a polar derivative of MK2 (2-cyano-3-[5-(9-ethyl-9H-carbazol-3-yl)-3,3,3,4-tetra-n-hexyl[2,2,5,2,5,2]-quater thiophen-5yl]), an organic molecule that has been used as an absorber in dye-sensitized solar cells^19^, and *R* is a functional group on the TMP. Zirconium phosphate was chosen, in part, due to previous experimental work that has demonstrated ordering of polar dyes using zirconium phosphate as a template^20^ in a manner similar to that proposed here. As described below, *M* and *R* are chosen to optimize the band alignment between the inorganic and organic layers. Atomic structure information for all systems in this work can be found in the Supplementary Information.

The MK2 molecule is modified to increase the polarity of the experimental structure. The large polarity of pyrimidine is exploited in this structure to create a highly polar MK2 molecule, as shown in the top of Fig. 2b. The band edges of MK2 are primarily composed of states from the four thiophene monomers in the central part of the molecule. The experimental HOMO-LUMO gap of this molecule is approximately 2 eV, although this can be modified by varying the length of the polythiophene chain^21^.

The polarity of the organic molecule is given by *P* = $\frac{D}{V}$, where *D* is the dipole of the molecule and *V* is the volume occupied by the molecule in the supercell. This polarization leads to an electric field, ${{\boldsymbol{E}}}_{{\boldsymbol{field}}}=\frac{{\boldsymbol{P}}}{2{{\boldsymbol{\epsilon }}}_{{\boldsymbol{r}}}{{\boldsymbol{\epsilon }}}_{0}}$, where ${\epsilon }_{r}$ is the relative permittivity of the organic molecule, ${\epsilon }_{0}$ is the permittivity of free space, and the factor of 1/2 is due to the void fraction of the MK2 molecule in the superlattice. This constant field imposes a rigid energetic shift between alternating inorganic layers. In order to separate excitons, we require this field to be greater than *E*_*b*_, the exciton binding energy in the organic material. While *E*_*b*_ has not been directly calculated or measured in this work, using well studied trends in organic molecules^22^, the binding energy in MK2 is estimated to be on the order of 0.5–1 eV.

To optimize the energy alignment of the organic and inorganic layers so that there are no barriers for electron (hole) transfer from the organic region into the inorganic electron (hole) conducting layer, we use relationships identified in our previous work to independently tune the band gap and the energies of the band edges in the alternating TMP layers^9^. In particular, large variations in the band gap can be obtained via in-plane cation substitution, while independent and precise control of the energies of the band edges is achieved through substitution of the out-of-plane *–R* functional groups. Through selection of cation substitution and *–R* functional group, the energetic barriers between the organic layer and the inorganic sheets can be minimized, maximizing *V*_OC_.

## Discussion

**Figure 3:**
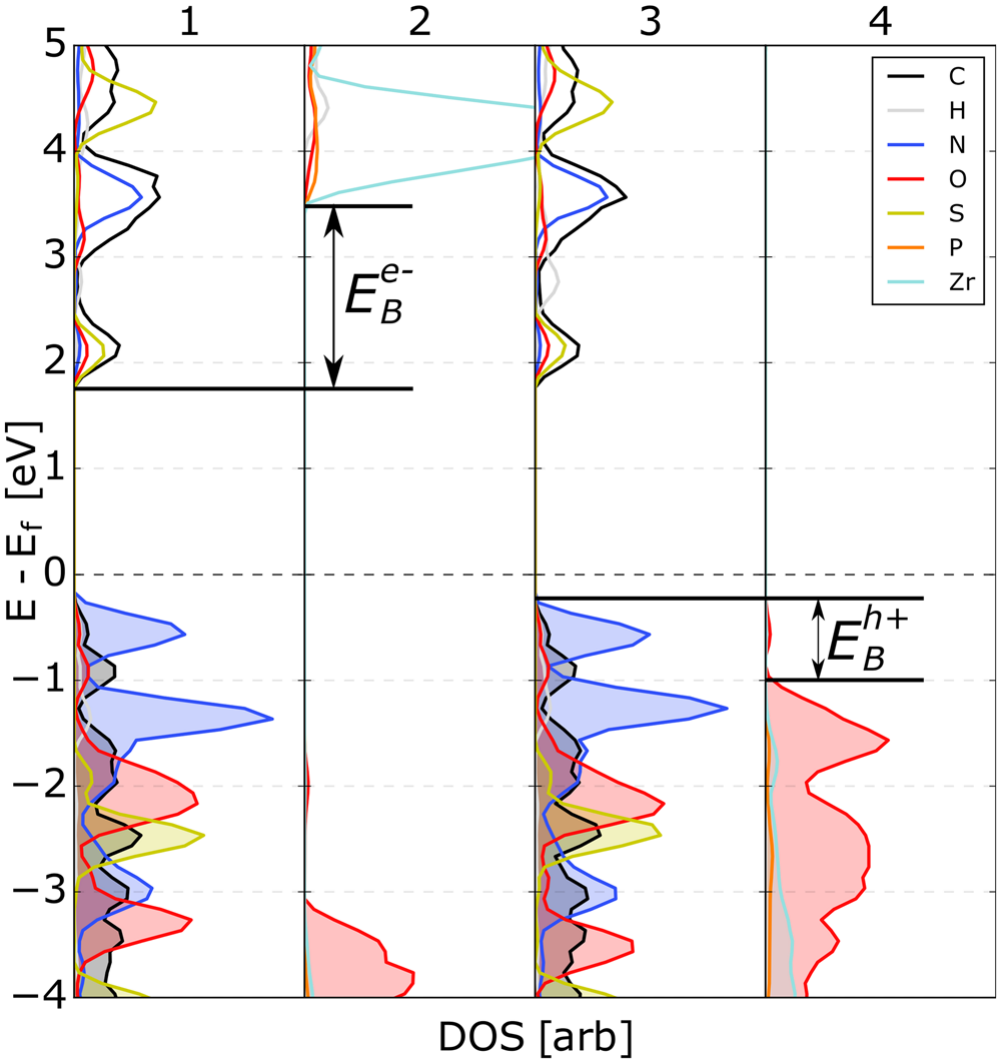
Atom-projected DOS of the TMP-organic superlattice with R = -OH and M = Zr. The DOS of the organic regions are shown in panels 1 and 3, while panels 2 and 4 show the TMP layers. In this case, the composition of adjacent inorganic layers are the same; the shift in the DOS from panel to 2 to 4 is due solely to the polarity of the organic molecules.

In Fig. 3, the projected density of states (pDOS) of the reference system with *R* = -*OH* and *M* = Zr is shown; in this case, the composition of all of the inorganic layers is the same. Creating a superlattice with organic molecules connecting this TMP sheet energetically shifts the CBM/VBM by 2.16 eV, as indicated by shift in the pDOS in panels 2 and 4 of Fig. 3. This gives an electric field across the MK2 molecule of 42.8 mVÅ^−1^. The isolated MK2 molecule was found to have a dipole of 7.95 D, creating a polarization of 1.032 μCcm^−2^, and the relative permittivity of thiophene is 2.8, giving an electric field $E=P/2\epsilon {\epsilon }_{0}$ = 38 mV/Å, in good agreement with that calculated from DFT. The small difference is likely a result of using the permittivity of thiophene as an approximation for that of the MK2 molecule. The exciton binding energy in polythiophene is 0.18 eV^23^, and typical binding energies in organic molecules are in the range 0.1–1 eV^22^; thus, the computed field will be sufficient to separate excitons in these materials.

As Fig. 3 shows, there is a large barrier of about 1.86 eV (${E}_{B}^{e-}$ in panel 2) for electron transfer from the conduction band edge of the organic regions (panels 1 and 3) into the conduction band edge of the inorganic electron transport layer in the reference system. There is also a barrier of 0.81 eV (${E}_{B}^{h+}$ in panel 4) for hole transfer from the valence band edge of the organic to the that of the hole conducting layer. Despite the electric field, these barriers will trap the free carries within the organic region. Extracting the charge carriers while concurrently maximizing *V*_OC_ requires that both ${E}_{B}^{e-}$ and ${E}_{B}^{h+}$ are close to zero.

**Figure 4:**
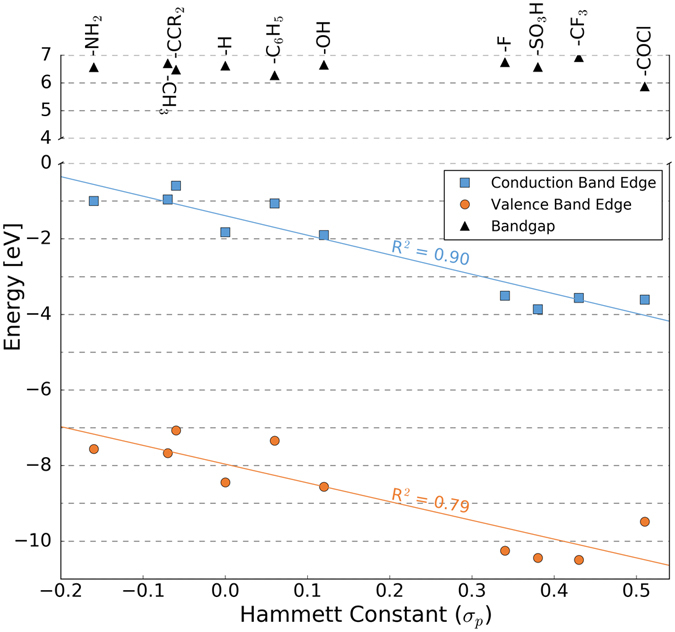
Linear relationships between band gap, electron affinity, and ionization potential and the Hammett constant of the functional group *R*.

To further tune the band alignment to achieve this requirement, we substitute the -OH groups on the surface of each of the Zr(HPO_4_)_4_ layers with different functional groups. Building off of previously demonstrated surface functionalization substitution^9^, we replace these -OH groups with a range of organic groups. Figure 4 shows the effects of *–R* substitution on the valence and conduction band edges of Zr(RPO_3_)_2_. As the figure shows, the energies of both the valence and conduction band edges vary linearly with respect to the Hammett constant^24^ (a measure of the electron withdrawing or donating character of a substituent often used in organic chemistry) of the functional group, while the magnitude of the band gap remains approximately constant for all -*R*.

To achieve the desired alignment of the valence band edge (VBE) of the organic absorber and TMP hole conducting layer, we require an upward shift of approximately 0.8 eV in the VBE of the latter. Referring to Fig. 4, this can be achieved by substituting the -OH functional group with -NH_2_. However, the barrier for electron transfer between the organic and TMP electron conducting layers is larger than the range of energies shown in Fig. 4. As we showed in previous work, we can systematically tune the band gap of the TMP layer by substituting some of the Zr with other transition metals^9^. Combining the observed trends for cation substitution and surface modification, we find that substitution of half of the Zr with Ti in addition to substitution of -OH with -F will shift the conduction band edge (CBE) of the electron conducting layer into good alignment with the CBE of the organic layer. We note that, due to the origins of the engineered band shifts, large amounts of exchange of the different *R* groups between the TMP layers will alter the predicted band alignment; however, low concentrations of *R* group disorder should not have a significantly affect the overall band alignment.

**Figure 5:**
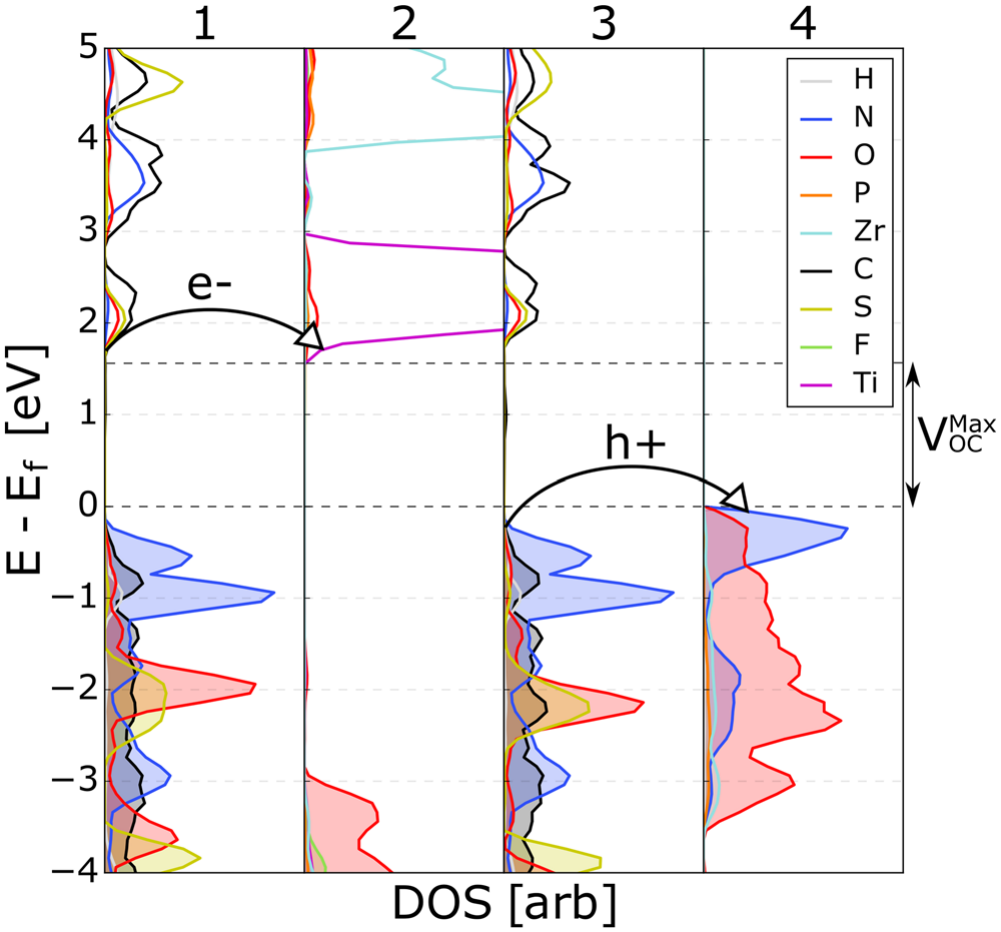
The pDOS of the different layers in the modified organic region. Panels 1 and 3 are the unmodified organic regions, panel 2 is ZrTi(FPO_3_)_2_, and panel 4 is Zr(NH_2_PO_3_)_2_. In this configuration, there is no hole barrier between panels 1,2 and 3,4 allowing charge extraction from the organic regions. The predicted V_OC,max_ of this system is 1.7 eV.

**Figure 6:**
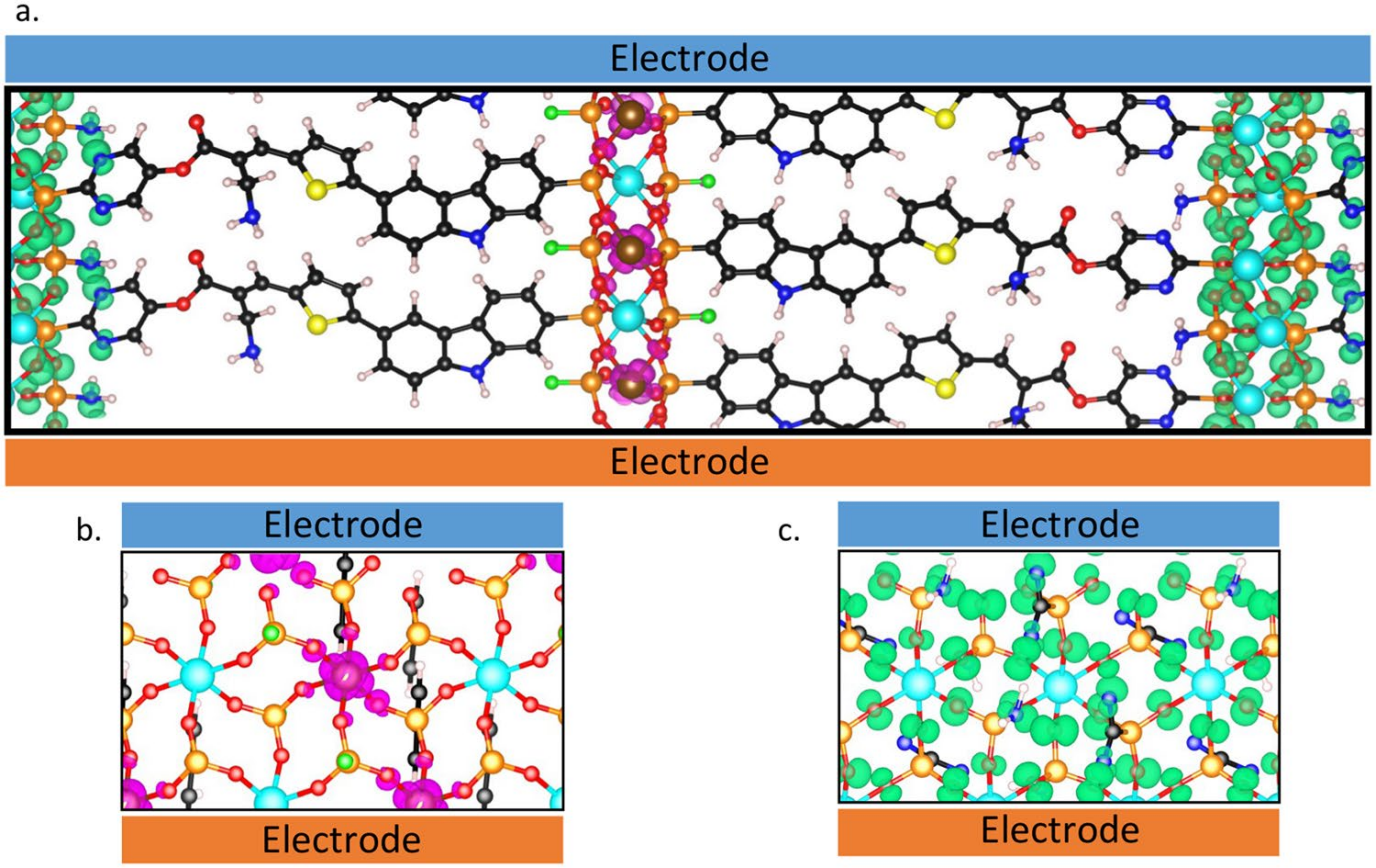
(**a**) The xz-plane of the bulk system showing spatial localization of the density of states at the conduction band edge (purple) and the valence band edge (green), states are highly localized outside the organic region, implying that the exciton is spatially separated in this system. (**b**) The *xy*-plane of the electron extracting layer and (**c**). The *xy*-plane of the hole extracting layer.

The overall bulk material, shown in Fig. 6, can be written as Zr_0.5_Ti_0.5_(*R*_0.5_F_0.5_PO_3_)_2_-Zr(*R*_0.5_(NH_2_)_0.5_PO_3_)_2_, where -*R* is the modified polar MK2 molecule. The pDOS of this system is shown in Fig. 5. The computed energy levels are in good agreement with the alignment predicted using the trends in band gap and VBE/CBE discussed above. We find that this system has a *V*_OC_ of 1.71 eV, a relatively small decrease with respect to the maximum possible value of 2 eV, the band gap of organic absorber. Excitons generated in the organic absorber (panels 1 and 3 in Fig. 5) are effectively separated due to the large electric field, and holes and electrons are driven to the relevant TMP layers (panels 2 and 4, respectively, in Fig. 5).

Figure 6, shows the states corresponding to the CBM and VBM of the system. As the figure illustrates, these states are spatially localized in alternating inorganic sheets, with little amplitude in the organic region. This further decreases the probability for electron-hole recombination. Using the computed density of states and the Boltzmann transport equation, we estimate the in-plane effective masses in the electron and hole conducting inorganic layers. We find that the in-plane effective mass in the electron conducting inorganic layer is 3.7, while the in-plane hole mass in the hole conducting layer is 4.2. Effective masses have been normalized by the mass of a free electron. Out-of-plane effective masses are 109.8 and 43.6 for the electron and hole layers, respectively, indicating free charge carriers will not be able to travel normal to their respective sheet, further preventing recombination. These values suggest these layers are sufficiently conducting to facilitate charge carrier extraction to the electrodes.

## Conclusions

In conclusion, we have presented a new approach for the design of hybrid organic-inorganic photovoltaics with low carrier recombination and large open-circuit voltage. We have demonstrated that layered transition metal phosphates provide a highly tunable framework for realizing such materials, and we have predicted a specific new material, based on zirconium phosphate covalently bound to a derivative of a known dye-sensitizing molecule, MK2, that forms an ordered organic-inorganic bulk superlattice with nanostructured dimensions.

We predict that this material has a large open-circuit voltage of 1.7 eV and, further, will exhibit low carrier recombination due to a combination of large local electric fields, nanoscale carrier diffusion distances, spatially localized conduction and valence band edge states, and relatively high mobility electron and hole conducting layers. This work suggests that hybrid TMPs are an exciting class of materials for further investigation in the search for achieving high efficiency, high power, and low cost photovoltaics.

### Computational Details

All structure optimization and electronic structure calculations were performed using the plane-wave DFT package Quantum Espresso (QE)^25^ using the GGA-PBE formulation of the exchange-correlation^26,27^, ultrasoft pseudopotentials^28^, and the Grimme van der Waals correction^29,30^. All pseudopotentials were obtained from existing QE libraries and tested by comparing computed and experimental structural properties of the relevant ground state elemental phases and bulk TMPs at standard temperature and pressure. All calculations were performed with an energy cutoff of 800 eV, and forces on all atoms were converged to less than 10 meV/Å. Ionization potentials and electron affinities were determined by referring the band energies of the 2D sheets to the energy of the vacuum using a 25 Å separation between periodic copies in the direction perpendicular to the plane of the 2D sheet. The GGA+*U* formalism^31^ was used in the hybrid TMP-organic systems. The value of *U* was chosen such that the band gap in the TMP region agreed with that computed for the bulk TMP using high accuracy GW calculations^9^. The polarity of isolated molecules was computed using the Quantum Chemistry package NWChem^32^. As GW calculations of the full hybrid organic-TMP system are computationally intractable, all band alignments were determined under the approximation of fixing the band gap of the organic region to the experimental value of 2 eV by shifting the CMB of the organic layer to replicate the experimental gap. Band edges in the inorganic layers were corrected in a similar fashion: CBM energies were rigidly shifted to the replicate the GW band gap. This approach was chosen as DFT is known to underestimate the energy levels of excited states in the system, while representing the occupied states accurately. No other changes were made to the nature of the DOS of the studied systems.

Conductivities were determined using the Boltzmann transport equations using the BoltzTraP^33^ code to compute the conductivity tensor in terms of the scattering time. As conductivities of TMPs are not well studied, the scattering time is not known. As such, this was used with the density of states to calculate the curvature of the band edges in terms of a free electron mass. This was verified through direct computation of the curvature of the band edges and using the effective mass approximation. Reported in-plane effective masses are the average of the along the in-plane principle directions (here as the crystallographic *a* and *b* directions). Out-of-plane curvatures are calculated normal to the plane of the TMP.

## Electronic supplementary material

Supplementary information
