# Publisher Correction: First-principles design of nanostructured hybrid photovoltaics based on layered transition metal phosphates

**DOI:** 10.1038/s41598-018-25541-2

**Published:** 2018-05-02

**Authors:** Levi C. Lentz, Alexie M. Kolpak

**Affiliations:** Department of Mechanical Engineering, Massachusetts Institute of Technology, 77 Massachusetts Ave, Cambridge, MA 02139 USA

Correction to: *Scientific Reports* 10.1038/s41598-017-01296-0, published online 28 April 2017

The original version of this Article contained an error in the Abstract.

“This work suggests that hybrid TMPs constitute an interesting class of materials for further investigation in the search for achieving high efficiency, high power, and low cost photo Zirconium phosphate was chosen, in part, due to previous experiment voltaics.”

now reads:

“This work suggests that hybrid TMPs constitute an interesting class of materials for further investigation in the search for achieving high efficiency, high power, and low cost photovoltaics.”

This error has now been corrected in the PDF and HTML versions of the Article.

