# Supplementary material for: First-principles design of nanostructured hybrid photovoltaics based on layered transition metal phosphates
Source: Sci Rep. 2017 Apr 28;7:1248. doi: 10.1038/s41598-017-01296-0 (PMC5430691; doi:10.1038/s41598-017-01296-0)
Supplement: Supplementary file 1 — Supplementary information [file 41598_2017_1296_MOESM1_ESM.pdf]

Supplemental Materials

January 23, 2017

1 Atomic positions for Figure 3

Figure 3 shows the projected DOS of a complex superlattice of alternating organic and inorganic materials. In Table 1, we report the Cell parameters and in Table 2 we report the crystallographic coordinates in this cell.

Table 1: Unit cell parameters for Figure 3. Length units are in Å and angle units are in degrees.

Parameter	Value
A	10.848
B	5.422
C	50.137
α	90.00
β	90.00
γ	120.00

2 Atomic Positions for Figure 5.

Figure 5 shows the projected DOS for the modified superlattice of Figure 1. In Table 3 shows the cell parameters and Table 4 shows the atomic positions.

Table 2: Atomic species for Figure 3. All coordinates are fractional.

Species	X	Y	Z	Species	X	Y	Z	Species	X	Y	Z	Species	X	Y	Z
N	0.440	0.268	-0.077	P	0.670	0.677	0.466	C	0.138	0.035	-0.304	H	-0.066	0.312	0.435
C	0.178	0.733	0.330	P	0.831	0.332	-0.035	C	0.329	0.337	-0.434	O	0.062	0.760	0.019
P	0.160	0.659	0.470	H	0.572	0.556	0.422	C	0.350	0.367	-0.329	C	0.498	0.616	-0.278
C	0.429	0.561	-0.205	C	0.240	0.724	0.159	C	0.151	0.659	0.434	H	0.756	0.651	0.422
C	0.227	0.289	-0.105	C	0.145	0.747	0.118	H	-0.072	0.394	0.114	Zr	0.504	0.015	0.499
H	0.142	0.298	-0.116	O	0.819	0.856	0.022	C	0.535	0.694	-0.251	C	0.324	0.399	-0.187
N	0.072	0.073	-0.174	O	0.609	0.361	0.473	N	-0.036	0.263	0.173	C	0.327	0.244	-0.118
C	-0.041	0.376	0.351	N	0.234	0.322	-0.079	H	-0.130	0.103	0.180	O	0.444	0.253	0.522
C	0.449	0.528	-0.419	C	0.678	0.703	0.430	H	0.770	0.067	0.577	C	0.688	0.709	0.070
C	0.138	0.665	0.380	C	0.132	0.027	-0.352	O	0.440	0.698	-0.149	O	0.392	0.657	0.522
C	0.271	0.855	0.420	H	-0.049	-0.256	-0.327	H	0.357	1.011	0.381	Zr	0.508	0.012	0.002
C	0.018	0.466	0.395	H	0.923	0.410	0.580	Zr	-0.004	-0.004	-0.003				
P	0.671	0.672	0.034	H	0.512	0.196	-0.114	O	0.570	0.778	0.025				
H	0.407	0.968	0.198	N	0.031	0.516	0.078	C	0.360	0.389	-0.281				
O	0.444	0.256	-0.019	O	0.821	0.860	0.477	H	-0.027	-0.043	-0.181				
P	0.344	0.354	-0.030	P	0.161	0.656	0.030	C	0.265	0.860	0.392				
O	0.683	0.149	-0.023	H	0.084	-0.048	-0.285	H	0.732	0.934	0.075				
O	0.106	0.349	0.478	H	0.707	0.921	0.424	C	0.433	0.233	-0.104				
O	0.398	0.669	-0.025	C	0.275	0.252	-0.352	C	-0.026	0.391	0.304				
Zr	-0.005	-0.006	0.502	H	0.460	0.541	-0.330	C	0.341	0.311	-0.066				
C	0.814	0.296	-0.071	O	0.893	0.652	0.529	H	0.193	-0.029	-0.201				
H	0.418	1.019	0.296	O	0.929	0.219	0.526	H	0.544	0.684	-0.430				
S	0.124	0.641	0.250	O	0.188	0.160	-0.022	C	0.286	0.835	0.234				
O	0.316	0.844	0.022	H	0.140	0.212	-0.212	C	0.023	0.462	0.423				
C	0.202	0.135	-0.422	H	-0.184	0.125	0.382	C	0.300	0.828	0.205				
C	0.317	0.318	-0.380	H	-0.058	0.357	0.158	S	0.279	0.270	-0.250				
H	0.535	0.668	-0.380	H	0.083	-0.045	-0.159	H	0.748	0.371	0.580				
C	0.042	0.468	0.194	H	-0.073	0.301	0.285	O	0.317	0.213	-0.146				
C	0.027	0.541	0.104	C	0.367	0.465	-0.159	O	0.103	0.340	0.024				
C	0.100	0.608	0.352	O	0.573	0.792	0.475	C	0.816	0.292	0.572				
O	0.315	0.864	0.478	H	0.760	0.634	0.077	C	0.061	-0.084	-0.328				
C	0.201	0.743	0.281	N	0.086	-0.042	-0.378	N	-0.090	0.297	0.377				
C	0.426	0.530	-0.233	H	-0.214	0.086	0.325	P	0.830	0.332	0.536				
C	0.197	0.670	0.187	O	0.611	0.355	0.027	H	0.354	1.120	0.113				
O	0.893	0.650	-0.028	H	0.504	1.120	0.246	H	0.569	0.725	-0.294				
H	0.529	0.730	-0.197	H	0.040	0.343	0.212	O	0.186	0.143	0.522				
P	0.339	0.348	0.531	H	0.286	0.912	0.331	O	0.057	0.755	0.480				
C	-0.106	0.267	0.327	H	0.583	0.580	0.079	H	0.772	0.072	-0.076				
N	0.268	0.914	0.077	C	0.197	0.129	-0.395	O	0.137	0.747	0.145				
C	0.281	0.259	-0.305	O	0.680	0.154	0.524	O	0.349	0.752	0.150				
H	0.637	0.867	-0.244	O	0.933	0.224	-0.026	H	-0.006	0.596	0.201				
C	0.116	0.623	0.305	C	0.155	0.697	0.066	C	0.180	0.151	-0.194				
H	-0.011	-0.201	-0.384	H	0.111	-0.008	-0.435	C	0.444	0.519	-0.392				
H	0.367	1.000	0.431	H	0.741	0.368	-0.078	C	0.393	0.983	0.252				
C	0.347	0.929	0.279	H	0.919	0.427	-0.080	C	0.263	0.942	0.104				

Table 3: Unit cell parameters for Figure 3. Length units are in Å and angle units are in degrees.

Parameter	Value
A	10.847
B	5.422
C	50.137
α	90.00
β	90.00
γ	120.00

Table 4: Atomic positions for Figure 5. All positions are in fractional.

Species	X	Y	Z	Species	X	Y	Z	Species	X	Y	Z
H	0.383	0.047	-0.178	O	0.423	0.341	-0.142	H	0.505	0.884	-0.199
C	0.215	0.889	0.389	C	0.044	0.218	0.351	O	0.593	0.868	0.471
O	0.320	0.760	0.031	H	0.153	0.029	-0.173	C	0.329	0.388	-0.436
H	0.221	0.894	0.326	O	-0.014	0.608	0.141	H	0.374	0.111	-0.212
C	0.261	1.038	0.247	C	0.343	0.146	-0.192	N	0.487	0.259	-0.073
O	0.606	0.319	0.036	H	0.738	-0.020	-0.068	P	0.366	0.318	-0.026
O	0.085	0.406	0.480	H	-0.048	-0.174	0.328	O	0.905	0.601	-0.032
O	0.930	0.228	0.525	O	0.431	0.256	0.520	O	0.831	0.894	0.480
N	0.157	0.853	0.076	C	0.143	0.618	0.378	P	0.848	0.387	0.529
O	0.079	0.873	0.476	N	0.188	0.018	-0.192	C	0.026	0.630	0.115
C	0.446	0.664	-0.206	H	0.021	0.203	0.434	C	0.272	0.305	-0.353
C	0.124	0.520	0.350	O	0.203	0.156	-0.026	C	0.277	0.322	-0.305
O	0.432	0.153	-0.014	C	0.165	0.662	0.326	N	0.013	0.134	0.378
H	0.343	1.074	0.288	O	0.118	1.078	0.153	C	0.249	0.124	-0.423
O	0.606	0.417	0.471	O	0.090	0.737	0.015	H	0.164	-0.092	-0.286
C	0.416	0.461	-0.187	Zr	0.518	-0.033	0.011	O	0.698	0.233	0.517
C	0.052	0.204	0.304	H	0.332	1.252	0.240	O	0.432	0.620	-0.015
N	0.004	0.349	0.077	H	0.272	1.120	0.426	H	0.430	0.844	-0.383
O	0.703	0.161	-0.011	H	0.086	-0.373	-0.327	C	0.380	0.645	-0.394
C	0.146	0.664	0.433	H	0.029	0.228	0.182	O	0.606	0.780	0.034
C	0.127	0.506	0.302	C	0.455	0.566	-0.159	O	0.922	0.152	-0.029
H	0.208	1.096	0.197	H	0.198	-0.074	-0.434	C	-0.089	0.414	0.192
O	0.095	0.280	0.022	Zr	0.508	0.058	0.496	C	0.100	0.594	0.065
C	0.419	0.778	-0.278	C	0.161	0.662	0.277	C	0.236	0.125	-0.395
C	0.154	0.831	0.231	S	0.061	0.512	0.249	N	0.167	-0.100	-0.378
C	0.333	0.484	-0.280	O	0.316	0.885	0.475	F	0.722	0.776	0.435
C	0.028	0.712	0.186	C	0.413	0.635	-0.233	H	0.713	0.670	0.083
C	0.050	0.827	0.159	H	0.380	0.694	-0.330	H	0.166	1.087	0.109
C	0.315	0.463	-0.330	P	0.702	0.645	0.037	H	0.141	-0.193	-0.197
C	0.073	0.371	0.394	O	0.506	0.811	-0.151	O	0.414	0.703	0.520
P	0.156	0.590	0.030	C	0.265	0.944	0.273	C	0.496	0.267	-0.100
H	0.454	0.846	-0.432	H	-0.144	0.421	0.211	C	0.428	0.377	-0.115
C	0.193	0.020	-0.305	C	0.130	0.883	0.204	H	0.122	-0.308	-0.383
Zr	0.006	-0.051	-0.006	H	-0.030	-0.069	0.384	N	0.782	0.724	0.067
H	0.449	0.927	-0.294	H	-0.169	0.340	0.176	P	0.687	0.738	0.466
C	0.302	0.384	-0.381	N	-0.030	0.230	0.197	H	0.028	0.082	0.285
P	0.157	0.708	0.468	C	0.148	-0.142	-0.328	H	0.651	0.163	-0.064
C	0.189	0.003	-0.352	P	0.342	0.392	0.529	C	0.216	0.911	0.417
C	0.120	0.876	0.101	C	0.009	0.058	0.328	Ti	0.011	0.056	0.500
C	0.394	0.646	-0.421	H	0.531	1.081	-0.245	C	0.358	0.497	-0.102
H	0.558	0.181	-0.109	N	0.347	0.486	-0.075	C	-0.032	0.370	0.101
N	0.746	0.167	-0.062	H	0.308	0.599	-0.113	H	-0.109	0.174	0.112
F	0.828	0.383	0.560	H	0.864	0.933	0.068	O	0.187	0.213	0.519
P	0.821	0.273	-0.032	H	0.269	1.079	0.377	C	0.407	0.360	-0.062
C	0.074	0.391	0.422	C	0.463	0.861	-0.251	H	-0.106	0.025	0.201
O	0.824	0.764	0.017	O	0.932	0.704	0.522	S	0.304	0.316	-0.250
